# Evaluation of 5-year imatinib treatment of 458 patients with CP-CML in routine clinical practice and prognostic impact of different BCR-ABL cutoff levels

**DOI:** 10.1002/cam4.59

**Published:** 2013-02-21

**Authors:** Hana Klamová, Kateřina Machová Poláková, Jan Mužík, Zdeněk Ráčil, Daniela Žáčková, Kateřina Steinerová, Michal Karas, Edgar Faber, Eva Demečková, Zuzana Michalovičová-Sninská, Jaroslava Voglová, Ľudmila Demitrovičová, Eva Mikušková, Elena Tóthová, Juraj Chudej, Imrich Markuljak, Eduard Cmunt, Jana Moravcová, Dana Dvořáková, Kyra Michalová, Marie Jarošová, Markéta Marková Šťastná, Petr Cetkovský, Ladislav DuŠek, Vladimír Koza, Marek Trněný, Karel Indrák

**Affiliations:** 1 Institute of Hematology and Blood TransfusionPrague, Czech Republic; 2 Institute of Biostatistics and Analyses, Masaryk UniversityBrno, Czech Republic; 3 Department of Internal Medicine Hematology and Oncology, University Hospital and Masaryk UniversityBrno, Czech Republic; 4 Department of Haematooncology, University Hospital PlzeňPlzeň, Czech Republic; 5 Department of Haematooncology, University Hospital OlomoucOlomouc, Czech Republic; 6 Department of Haematology and Blood Transfusion, University Hospital BratislavaBratislava, Slovak Republic; 7 2nd Department of Internal Medicine, Division of Hematology, University Hospital Hradec KrálovéHradec Králové, Czech Republic; 8 Department of Internal Medicine, Division of Haematology and Blood Transfusion, National Cancer Institute BratislavaBratislava, Slovak Republic; 9 Department of Hematology and Oncohematology, L. Pasteur University and UPJSŠKoŠice, Slovak Republic; 10 Department of Haematology and Blood Transfusion, University Hospital MartinMartin, Slovak Republic; 11 Department of Haematology, FD Roosevelt University Hospital and Health Care CentreBanská Bystrica, Slovak Republic; 12 1st Department of Internal Medicine, General University HospitalPrague, Czech Republic

**Keywords:** BCR-ABL ratios, CML, ELN definitions, imatinib

## Abstract

We evaluated responses to the treatment and long-term outcomes of chronic myeloid leukemia patients treated with imatinib as first-line treatment in routine clinical setting from two countries with centralized tyrosine kinase inhibitors (TKIs) treatment. We assessed prognostic significance of European LeukemiaNet (ELN) 2006- and 2009-defined responses and the prognostic value of molecular responses at defined time points on 5-year survivals. Among the cumulative rates of incidence of hematologic, cytogenetic, and molecular responses and all important survival parameters, we evaluated the prognostic significance of different BCR-ABL transcript-level ratios (≤1%; >1%–≤10%; >10%) at 3, 6, 12, and 18 months (*n* = 199). The ELN optimal response criteria and their predictive role were significantly beneficial for event-free survival at all given time points. We found significant improvement in survivals of patients with BCR-ABL lower than 10% in the 6th and 12th months. Significantly better outcome was found in patients who achieved major molecular response (MMR) in the 12th month. The cumulative incidences of complete cytogenetic response (CCyR) and MMR were significantly associated with the molecular response in the 3rd month. The ELN response criteria and their predictive role were helpful at given time points; however, the 2009 definition did not significantly alter the prognostic accuracy compared with that of the 2006 definition. The significant value was observed for cytogenetic responses at the 6th and 12th month. Moreover, progression-free and event-free survivals were improved with MMR at the 12th month.

## Introduction

Imatinib (IM; originally STI571), a BCR-ABL tyrosine kinase inhibitor (TKI), is a highly potent targeted therapeutic agent that has substantially changed the treatment of patients with chronic myeloid leukemia (CML). For patients with newly diagnosed disease in the chronic phase (CP), it markedly improves prognosis 1,2. Following the IRIS multicenter clinical trial (International Randomized Study of Interferon vs. STI571), which demonstrated an estimated 8-year overall survival (OS) of 85%, progression-free survival (PFS) of 93%, and event-free survival (EFS) of 81%, IM became the first-choice medication for CML patients in the CP 3,4. The European LeukemiaNet (ELN) recommendations, initially published in 2006 5, aimed to rationalize CML treatment, so as to unify treatment procedures and to optimize the frequency and types of laboratory analysis. This first published summary of recommendations for CML treatment and monitoring particularly focused on early detection of its failure 5. ELN 2009 is an updated version that reflects the experience with second-generation TKIs and the long-term outcome data with the aim of managing the therapy for survival maximization and normal quality of life 6. According to the ELN recommendations, the response to first-line IM can be stratified according to the therapeutic response at defined time points 5,6, where optimal responders were likely to reach long-term benefit from the treatment, in contrast to the others. One of the important changes in the upgraded version was the definition of the 3rd month optimal response. At least minor cytogenetic response was introduced next to the complete hematologic response, both considered as an optimal response achievement in the 3rd month 6. Outside clinical trials, there is still lack of data on the impact of IM on patient outcome as well as on the applicability of ELN recommendations to clinical practice. As the treatment of patients in routine clinical practice is influenced by many factors not encountered in clinical trials, the extrapolation of procedures and recommendations from clinical trial results to clinical practice may not be straightforward. For this reason, it is necessary to study the experience and outcomes from the routine practice.

BCR-ABL transcript-level monitoring (BCR – gene encoding the break point cluster region protein; ABL – Abelson murine leukemia viral oncogene homolg 1) is a highly useful diagnostic tool that controls the effectiveness of the CML treatment and indicates at an early stage resistance development or disease progression. So the kinetics of BCR-ABL transcript level is very important and many reports proved its usefulness for disease management e.g., 7–9. However, it is still a matter of contention if BCR-ABL transcript-level data observed in the defined time points may be significantly predictive for the long-term outcome of CML patients treated with IM first line and might improve or even replace the prognostic significance of cytogenetic data 10,11. Major effort was put into the interlaboratory harmonization and conversion factors (CF) calculation and their validation at international scale (IS) 12,13. Many labs across Europe have obtained their validated CF, but the international study showed the impending instability of the CF within one single lab 14. Therefore, even estimated and validated CF could not guarantee that laboratories will perform the monitoring in an entirely comparable way, because new causes that may contribute to an increase in the variability of the measurements may appear over time (e.g., other sources of chemicals, another batch, upgraded instrumentation, human factor).

In the Czech Republic and Slovakia (regions with altogether ~15 million inhabitants), the TKI treatment of CML patients is centralized in 13 major hemato-oncologic centers. Treatment data from all these centers are collected in two databases: CAMELIA 15 and INFINITY 16 including all patients treated with IM. The patients are closely monitored and treated according to the ELN recommendations 5,6. In this study, we focused on analysis of the prognostic value of the ELN 2006- and 2009-recommended responses evaluations for the first-line IM treatment. Moreover, we attempted to evaluate the prognostic significance of different cutoffs of BCR-ABL transcript level in the 3rd, 6th, 12th, and 18th month in the outcomes and compare data with the recently reported results from the IRIS study.

## Design and Methods

### Patients

Data from a cohort of 458 unselected patients with newly diagnosed CML in the CP, treated with first-line IM in 11 Czech and Slovak hemato-oncologic centers between the years 2003 and 2009 were analyzed. The databases **CAMELIA** (**C**hronic **M**y**E**loid **L**eukem**IA)** and **INFINITY** (tyrosine kinase **I**nhibitors i**N FI**rst a**N**d follow**I**ng CML **T**reatment) collected anonymized data of 306 and 152 patients, respectively, with approval of the ethic committees and patients' informed consents.

### Definitions of treatment responses and the endpoints

Treatment responses were evaluated according to the ELN recommendations released in 2006 and 2009 5,6. We assessed the cumulative incidence rates of complete hematologic response (CHR), major cytogenetic response (MCyR), complete cytogenetic response (CCyR), major molecular response (MMR), and complete molecular response (CMR).

OS was defined as the time from the start of IM administration to death from any cause, irrespective of IM discontinuation. Survival to CML-related death (OS_CML_) was defined as the time to death due to CML only. Transformation-free survival (TFS) was defined as survival without evidence of accelerated phase (AP) or blast crisis (BC) or death from any cause during IM therapy. PFS was defined as in the IRIS trial 17, that is, survival without evidence of AP or BC, loss of CHR, loss of MCyR, increased white blood cell count (in patients who had never had CHR), or death from any cause while on IM treatment, whichever came first. Events EFS were defined as a progression (as in PFS described above), loss of CCyR together with improved definition including failure to achieve CHR at 6 months, MCyR at 12 months, and CCyR at 18 months, or intolerance of IM as the cause for discontinuation, whichever came first 5,18. Alternative treatment-free survival (ATFS) was defined as the time since start of IM to change to any alternative treatment or death from any cause during the IM therapy 16. ATFS reflected the real proportion of patients who stayed on IM despite the event occurrence.

Cytogenetic and molecular analyses were performed according to ELN recommendations 5,6. Conventional cytogenetic analysis used the G-banding technique, and at least 20 metaphases were analyzed.

### Evaluation of prognostic significance of the ELN recommendations

Based on the quality of a response to IM at defined time points (3, 6, 12, and 18 months) determined using the ELN 2006 recommendations, the patients were stratified into the following categories: optimal response, suboptimal response, and treatment failure 5. Subsequently, the prognostic impact of optimal and less than optimal responses on TFS, PFS, and EFS was assessed. On comparing the ELN 2009 recommendations with the 2006 version, the treatment response at 3 months is more strictly defined: optimal response = CHR and at least a minor CyR (mCyR); suboptimal response = no CyR; and treatment failure = no CHR 6. Impact of the changes between these two editions (2006 and 2009) on survival end points was assessed on a subset of 156 patients, in whom the cytogenetic analysis was performed in the 3rd month.

### Prognostic significance of molecular response

Patients' survival was evaluated according to different rates of BCR-ABL transcript level at 3, 6, 12, and 18 months. BCR-ABL transcript quantity data were considered only from a subset of 199 patients, whose samples were analyzed in three laboratories with the standardized quantitative real-time reverse transcription-polymerase chain reaction (RT-PCR) methodology at the time of data collection. These three laboratories were annually controlled by the external quality control organized by the National reference laboratory for DNA diagnostics in the Czech Republic (accredited by the Czech Accreditation Institute; http://www.cia.cz/default.aspx?id=45) and produced comparable results. In the meantime, the laboratories have started their participation in the international BCR-ABL standardization project (EUTOS for CML) 12; however, any CFs for the calculation into the IS had not yet been and recently observed CF should not be applied retrospectively. We were aware that the interlaboratory comparison was not absolute, but we intended to evaluate the molecular data as these reflect the clinical practice that had been running during the years 2003–2009.

An optimized multiplex RT-PCR was adapted from the method of Cross et al. to determine the type of BCR-ABL transcript 19. Quantitative real-time RT-PCR was performed according to Europe Against Cancer (EAC) recommendations 20, using *ABL* (two laboratories) or *B2M* (one laboratory) as control genes. The MMR was identified if the BCR-ABL transcript at any levels was stably ≤0.1%. BCR-ABL-negative sample (CMR) was identified if the BCR-ABL transcript was stably undetectable by quantitative real-time RT-PCR and/or nested RT-PCR 6. Patients with nonstable MMR or CMR were excluded from evaluations.

### Statistical methods

The frequency tables and standard descriptive statistics (mean, median, minimum, maximum) were used to summarize patient characteristics. The probabilities of OS, OS_CML_, TFS, ATFS, PFS, and EFS, were estimated using the Kaplan–Meier method. The probabilities of hematological, cytogenetic, and molecular responses were estimated using the cumulative incidence method. The point estimates were supplied with 95% confidence intervals (CI). Landmark analysis of TFS, PFS, and EFS was performed based on treatment responses according to ELN criteria 5,9. Univariate analyses estimating prognostic power of treatment response for TFS, PFS, and EFS were based on log-rank test. Level of statistical significance α = 0.05 was used in all analyses. Analyses were performed by using statistical software SPSS 12.0.1 for Windows (IBM Corp., Armonk, NY) and STATISTICA 8.0 for Windows (StatSoft, Tulsa, OK).

## Results

### Patient characteristics and treatment

Between July 2003 and July 2009, a total of 458 adult patients (median age 52 years (range 17–81), men 51.3%) with Ph-positive CML in the CP (one patient was BCR-ABL positive, but without Ph chromosome), treated with IM as a first-line therapy, were recorded in the databases CAMELIA and INFINITY (Table 1). Median follow-up on IM treatment was 33.1 months (range 1.4–82.1); median time from diagnosis to start of IM therapy was 1.2 months (range 0–13.3). Initially administered daily dose of IM was 400 mg. The dose was reduced in 131 (28.6%) patients, mainly because of side effects (e.g., vomiting, diarrhea, headache, hematologic toxicity), and escalated during the treatment to 600–800 mg/day in 101 patients (22.1%) mainly because of suboptimal response. IM was permanently discontinued in a total of 112 (24.5%) patients after median 14.4 months (range 0.2–25.7) from the start of therapy. Reasons for the discontinuation included disease progression or IM failure (*n* = 54), intolerance to IM (*n* = 30), elective allogeneic transplantation (*n* = 14), death from non-CML-related causes (*n* = 8), and other reasons (*n* = 6).

**Table 1:** Characteristic of patients and treatments (*n* = 458)

Characteristics	*N* (%)
Female/male	223/235
(48.7/51.3)
ECOG performance	209/175/39/1/34
0/1/2/3/NA	(45.6/38.2/8.5/0.2/7.4)
Sokal risk group	185/169/101/3
Low/Intermediate/High/NA	(40.4/36.9/22.1/0.7)
Hasford risk group	183/210/62/3
Low/Intermadiate/High/NA	(40.0/45.9/13.5/0.7)
Add. chromosomal abnormalities in Ph+ cells	37/331/90
Yes/No/NA	(8.1/72.3/19.7)
Type of BCR-ABL transcript	272/139/9/38
(b2a2/b3a2/other^1^/NA)	(59.4/30.3/2.0/8.3)
Palpable spleen	219/237/2
Yes/No/NA	(47.8/51.7/0.4)
	Median (range)
	
Age (years)	52 (17–81)
Interval since diagnosis to start of IM therapy (months)	1.2 (0–13.3)
Leukocytes (10^9^/L)	71.5 (3.3–612)
Platelet count (10^9^/L)	414 (35–3308)
Hemoglobin (g/L)	123 (115–170)
Basophiles in PB (%)	4 (0–21)
Blasts in PB (%)	1 (0–10)
Follow-up – all patients	33.1 (1.4–82.1)
Follow-up – alive patients	34.2 (5.7–82.1)
	
Treatment	*N* (%)
First dose	
<400 mg/day	55 (12.0)
400 mg/day	382 (83.4)
>400 mg/day	21 (4.6)
Dosage changes during treatment	
<400 mg/day any time	131 (28.6)
>400 mg/day any time	101 (22.1)
400 mg/day only all the time	249 (54.4)
Treatment interruption	9 (2.0)
Permanent discontinuation of imatinib treatment	112 (24.5)
Progression or failure of treatment	54 (11.8)
Intolerance of imatinib treatment	30 (6.6)
Targeted transplantation	14 (3.1)
Other reason	6 (1.3)
Death from non-CML-related cause	8 (1.7)
Time to permanent discontinuation of imatinib treatment (*N* = 112)	Median (range)
Months	14.4 (0.2–57.7)

1 Eleven patients (2.4%) have two types of BCR-ABL transcripts.

### Treatment responses and survival end points

Estimated OS at 5th year was 90.2% (CI 86.5–93.8%), OS_CML_ was 96.6% (CI 94.6–98.5%), TFS was 93.9% (CI 90.9–96.9%), PFS was 80.7% (CI 75.2–86.3%), EFS was 58.8% (CI 49.6–68.0%), and ATFS was 61.8% (CI 53.7–69.9%).

The cumulative incidences of hematologic and cytogenetic responses among 458 patients are illustrated in Figure S1a and summarized in Table S1. Cumulative incidences of MMR and CMR (Fig. S1b, Table S1) were evaluable in the cohort of 199 patients (see Methods). In line with the treatment duration, the number of patients who achieved CCyR and MMR rose continuously, with a rise in CCyR from 61.7% after 18 months to 79.2% after 5 years of IM treatment, and in MMR from 51.2% to 71.8%. BCR-ABL negativity increased from 11.3% after 18 months to a predicted 37.0% after 5 years of IM.

### Prognostic significance of optimal response defined by ELN 2006 5

The prognostic significance of achieved optimal versus nonoptimal responses on the 5-year probability of survival without transformation, progression, and event is summarized in Table 2. Optimal responses at 6 months (partial cytogenetic response [PCyR]) and 12 months (CCyR) were predictive of PFS (*P* = 0.041, *P* = 0.021) and EFS outcomes (*P* = 0.001, *P* = 0.001) after 5 years of IM therapy. Optimal response at 3 months was predictive of EFS. The 18-month interval defined according to ELN was not predictive of TFS, PFS, and EFS.

**Table 2:** Prognostic significance of optimal response

Landmark	Optimal response definition	TFS^2^	PFS^2^	EFS^2^
3 months (Blood 2006)^1^	CHR	0.017	NS (0.148)	0.024
3 months (JCO 2009)^1^	CHR + mCyR	0.005	NS (0.116)	0.007
3 months (Blood 2006)	CHR	<0.001	NS (0.079)	<0.001
6 months	PCyR	NS (0.245)	0.041	0.001
12 months	CCyR	NS (0.745)	0.021	<0.001
18 months	MMR	NS (0.296)	NS (0.179)	<0.001

TFS, transformation-free survival; PFS, progression-free survival; EFS, event-free survival; IM, imatinib; AP, accelerated phase; BC, blast crisis; CHR, complete hematologic responses; MCyR, major cytogenetic response; PCyR, partial cytogenetic response; CCyR, complete cytogenetic response; MMR, major molecular response; NS, not significant.

1 Subgroup of patients with known cytogenetic status in 3rd month.

2 In this study, TFS was defined as survival without evidence of AP or BC or death from any cause during IM therapy. PFS was defined as survival without evidence of AP or BC, loss of CHR, loss of MCyR, increased white blood cell count (in patients who had never had CHR), or death from any cause while on IM treatment, whichever came first. EFS was defined as a progression (as in PFS described above), loss of CCyR together with improved definition including failure to achieve CHR at 6 months, MCyR at 12 months, and CCyR at 18 months, or intolerance of IM as the cause for discontinuation, whichever came first 5,18.

### Probability of survival according to optimal response in the 3rd month: comparison of ELN definitions 2006 5 and 2009 6

**Figure 1:**
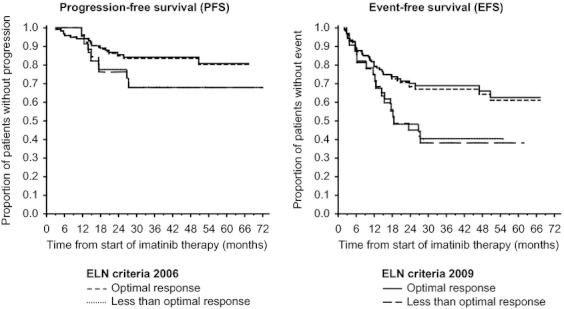
Effect of optimal versus nonoptimal responses on PFS and EFS: comparison of ELN 2009 and 2006 criteria (3rd month response). In this study, PFS was defined as survival without evidence of AP or BC, loss of CHR, loss of MCyR, increased white blood cell count (in patients who had never had CHR), or death from any cause while on IM treatment, whichever came first. EFS was defined as a progression (as in PFS described above), loss of CCyR together with improved definition including failure to achieve CHR at 6 months, MCyR at 12 months, and CCyR at 18 months, or intolerance of IM as the cause for discontinuation, whichever came first 5,18. PFS, progression-free survival; EFS, event-free survival; ELN, European LeukemiaNet; AP, accelerated phase; BC, blast crisis; CHR, complete hematologic responses; MCyR, major cytogenetic response; IM, imatinib; CCyR, complete cytogenetic response.

We analyzed the probability of survival in patients who achieved optimal response in the 3rd month according to both the ELN 2009 (achievement of CHR and mCyR) and the ELN 2006 recommendations (achievement of CHR) in comparison with nonoptimal responders. Moreover, we evaluated the impact of the definition “improvement” released in 2009. The number of evaluable patients who were examined cytogenetically in 3rd month was 156. The 3rd month optimal response examination according to both the ELN 2006 and ELN 2009 showed significantly higher probability to survive without EFS, while it was not significant for TFS and PFS after 5 years of IM treatment (Table 2). Figure 1 shows no significant difference between the ELN 2009 and 2006 classifications of optimal responders in the probability to survive without progression, and event.

### Survival of patients according to BCR-ABL transcript levels

Only BCR-ABL molecular data from 199 patients that had been obtained from laboratories with standardized and comparable methodologies were considered (see Methods). The 5-year probability of transformation-free, progression-free, and event-free survivals were calculated according to BCR-ABL transcript levels (≤1%; >1%–≤10%; >10%) in 3rd, 6th, 12th, and 18th months. The 6th month landmark showed significant differences between the group with BCR-ABL transcripts higher than 10% and the groups with the levels equal to or lower than 10% for PFS and EFS (Table 3). A significant difference was found between the groups with BCR-ABL level higher than 1% and those with levels equal to or lower than 1% in the 12th month. Even more significantly higher probability of PFS and EFS was found in patients who achieved MMR in comparison with patients with BCR-ABL level higher than 0.1%. The 18th month landmark showed significantly higher probability to survive without an event in patients who achieved MMR. The 3rd month landmark was not significant when comparing groups that achieved different BCR-ABL transcript levels for the TFS, PFS, and EFS after 5 years.

**Table 3:** Probability of survivals according to BCR-ABL transcript ratios. Patients in whom the progression or event occurred before the point of evaluation or have a shorter follow-up are not included in landmark analysis. BCR-ABL transcript ratios in the 3rd month were not significant for TFS, PFS, and EFS. The 6th, 12th, and 18th month landmark was not significant for TFS

Transcript ratio	Landmark					
6th month		12th month		18th month	
PFS	EFS	PFS	EFS	PFS	EFS
≤1.0% (*n*)	77	77	106	100	70	68
>1.0% and ≤10% (*n*)	38	37	24	23	11	10
*P* level	NS	0.057	0.002	<0.001	NS	0.008

≤1.0% (*n*)	77	77	106	100	70	68
>10% (*n*)	28	27	12	11	11	7

*P* level	0.013	<0.001	0.042	<0.001	0.007	<0.001
>1.0% and ≤10% (*n*)	38	37	24	23	11	10
>10% (*n*)	28	27	12	11	11	7

*P* level	0.048	0.045	NS	0.004	NS	NS
≤0.1% (*n*)	30	30	63	61	46	45
>0.1% and ≤1.0% (*n*)	47	47	43	39	24	23

*P* level	NS	NS	0.041	NS	NS	NS
MMR (*n*)^1^	30	30	63	61	46	45
no MMR (*n*)^1^	113	111	79	73	46	40
*P* level	NS	NS	0.003	<0.001	NS	0.008

TFS, transformation-free survival; PFS, progression-free survival; EFS, event-free survival; MMR, major molecular response.

1 Cumulative achievement of MMR till landmark time.

### Cumulative incidence of CCyR and MMR according to BCR-ABL transcript levels in the 3rd month

**Figure 2:**
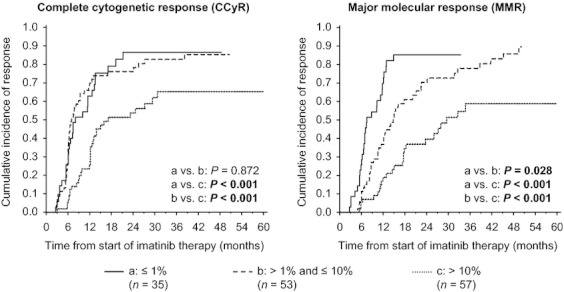
Cumulative incidence of CCyR and MMR according to BCR-ABL level in 3rd month. CCyR, complete cytogenetic response; MMR, major molecular response.

The probability of cumulative incidence of CCyR in 12th month and MMR in 18th month was analyzed according to the BCR-ABL transcript level with defined ranges in 3rd month. Again, only reliable BCR-ABL data that were available from 145 patients in the 3rd month were considered in this analysis. A significantly higher probability to achieve CCyR at 12th month was found for the group with BCR-ABL quantity in the 3rd month lower than or equal to 10% than in the group with BCR-ABL higher than 10% (*P* < 0.001) (Fig. 2A, Table S2). Patients with BCR-ABL transcript level >10% in the 3rd month had a significantly lower probability to achieve MMR, compared with those with lower levels (*P* < 0.001). Significant differences were found even between the groups with BCR-ABL transcript levels ≤1% and those with levels >1% to ≤10% (*P* = 0.028) (Fig. 2B, Table S2). An increase in the cumulative incidence of CCyR and MMR after 4 years and 30 months on IM treatment, respectively, was found in all three BCR-ABL groups (Table S2).

## Discussion

Following the results of the IRIS multicenter trial 3,4, IM promptly became the standard frontline therapy of CML in CP. Some single-center reports on the use of IM in clinical practice have been published, but further evaluations of data from nonselected cohorts of patients or population-based studies are required.

This study is focused on nonselected cohort of CML patients in CP treated in clinical practice with IM as first-line therapy during the years 2003–2009. Recalculated for the whole population in both countries (Czech Republic and Slovakia ~15 million inhabitants), annual incidence rate corresponds to 0.78 CP CML treated with IM first line per 100,000 adults. According to Rohrbacher et al. 21 the incidence rate of CML varies from 0.6 to 2.0 cases per 100,000, higher in men than in women, which is in agreement with our cohort. The access to IM first line by health-insurance companies in 2003–2004 was limited, and part of elderly patients were not referred to the hematologic centers; this may explain the observed lower age median in this study than expected 22.

The direct comparison of data from clinical practice with data from clinical trials may be difficult. The problem lies in different cohorts, survival definitions, and follow-up 16,23. Moreover, the definitions of end points can differ even in the updates of a specific study 3,24. However, regarding the probabilities of OS, PFS (in our study as TFS), EFS (in our study as PFS), the presented multicenter data of nonselected cohort of 458 patients are highly similar to the IRIS study 3, with the intention-to-treat analysis from Hammersmith hospital 18, and with our previous study on patients from two centers 16. Among OS, TFS, and loss of CHR, we may directly compare the observed probabilities of PFS (survival without evidence of AP or BP, loss of CHR, loss of CyR, increased WBC (in patients who had never had CHR), or death from any cause while on IM treatment, whichever came first); this definition was reported as EFS in the mentioned studies showing 83% probability at 7 years in IRIS, 81.3% at 5 years in Hammersmith, and 78.1% at 4 years in INFINITY in comparison with this report showing 80.7% at 5 years. Among the survival analyses (“time to event analyses”), we used a recently published parameter ATFS, that is, the indicator of survival without the administration of an alternative treatment 16, which is, in our opinion, an improved definition for the characterization of the proportion of patients who will continue on IM treatment despite the events. This is supported by our results showing that 69.7% (95% CI: 64.2–75.2) of patients will be still treated with IM after 4 years from treatment initiation (when EFS = 66.6% [95% CI 60.6–71.4]). It is important to note that second-generation TKI has been approved in both countries since 2007; therefore, the ratio of patients who stayed on IM in spite of an event is quite high for the treatment between the years 2003 and 2009.

### Prognosis of ELN-defined responses

As the next goal, we evaluated the prognostic significance of treatment responses defined by ELN 5,6. A significantly better prognosis for PFS was demonstrated for optimal response to IM (i.e., cytogenetic response) at 6 and 12 months, which is in agreement with other recent reports 25,26,27. For EFS, including 3rd month, all three evaluated prognostic time points were significant.

The optimal response in the 18th month is according to ELN defined as MMR achievement. In spite of EFS, we did not find significant differences in the probability to survive without progression between patients with and without MMR in the 18th month.

On the subgroup of patients (*n* = 156), we showed that the optimal response defined for the 3rd month according to 2006 and 2009 ELN recommendation was significantly associated with better survival. Additionally, an achievement of mCyR after 3 months of the treatment (ELN 2009) did not significantly improve the survival prognosis over that based solely on CHR (ELN 2006). In contrast, MCyR in the 3rd month was significantly associated with 5-year PFS (defined as TFS in our study) in Hanfstein et al. 10.

### Prognosis of BCR-ABL transcript levels in defined time points

Currently, a frequently discussed topic in CML treatment is the quickness to achieve deep molecular response after TKI treatment initiation and its prognostic impact. It was postulated and shown in some works that the earlier and deeper the molecular response was, the more likely the response to treatment would be better and longer lasting 28.

In this study, we were able to analyze BCR-ABL molecular data from a subset of 199 patients who were monitored in the three laboratories that were in the meantime standardized and compared with each other. BCR-ABL monitoring was performed in those patients regularly at least every 3 months including defined time points such as the 3rd, 6th, 12th, and 18th months after start of IM therapy. We found that patients were divided nearly equally into the three groups according to achieved BCR-ABL transcript level 3 months after IM start: ≤1%; >1% ≤10%; >10%. However, among these groups, we did not find any significant predictive value for survivals without transformation, progression, and event. Recent works of Hanfstein et al. 10 and Marin et al. 11 proved that the 3rd month BCR-ABL transcript level higher than 10% IS was significantly predictive of survival without progression (i.e., survival without evidence of AP or BC or death from any cause during IM therapy) on IM first line. A cohort of patients of similar size to the one used in our study was the Hammersmith cohort of 282 patients; however, their OS survival was 84.3% in the 8-year probability, allowing better discrimination in comparison with our study when the outcome of our patients was better in the 5-year probability (OS 90.2% and OS_CML_ 96.6%). The OS in our study was comparable to Hanfstein et al. 10; however, the cohort of patients of the German Study VI was 3.5 times larger. We suppose that longer follow-up and larger cohort of patients in our study are needed to showing BCR-ABL data in the 3rd month landmark predictive for outcome.

Hanfstein et al. 10 and Marin et al. 11 showed an impact of BCR-ABL equal to or lower than 1% IS cut-off in the 6th month on significantly better survival. Significant differences were found in more detailed definitions of PFS and EFS in our study within the BCR-ABL groups after 6 and 12 months of IM therapy (exception was for PFS between ≤1.0% vs. >1.0% –≤10% in the 6th month). No benefit was found in PFS or EFS for patients with MMR in the 6th month. This observation is consistent with the data published by Hughes et al. 17 showing no significant difference in EFS (defined in our study as PFS) on comparing MMR versus no MMR achievement and versus >0.1% to ≤1% in the 6th month landmark.

MMR achievement in the 12th month showed significantly higher probability of PFS and EFS in comparison with patients without MMR. This is in agreement with the recently published data from IRIS study 17 and Marin et al. 11, which confirmed better EFS (in our study defined as PFS) in patients with MMR in the 12th month. In spite of IRIS and Jabbour et al. 26, we found a significant difference for PFS even when comparing MMR versus >0.1%–≤1.0%. Hehlmann et al. 25 proved better PFS (defined as survival free of AP and BC) significantly associated with MMR in the 12th month, which we did not confirm in our study for TFS (i.e., survival free of AP, BC, or death from any cause during IM therapy).

To explore the possible importance of depth of early molecular response, we investigated the cumulative incidence of MMR and CCyR according to the BCR-ABL transcript level in the 3rd month. This analysis clearly showed that patients with a BCR-ABL ratio >10% had a significantly lower probability of achieving MMR and CCyR than those with lower levels (*P* = 0.001). The greatest reduction in BCR-ABL within the first 3 months of IM therapy was significantly associated with the cumulative incidence of CCyR and MMR optimal achievements in the 12th month and the 18th month, respectively. Our results are consistent with previous work showing that the deeper the molecular response and the earlier these responses are achieved, the higher is the probability of achieving CCyR and MMR 10,11,27. Additionally, irrespective of optimal response definition for CCyR and MMR achievement, the 4-year and 30-month cumulative incidence of CCyR and MMR, respectively, showed that there is still a chance that significant proportion of patients will achieve required responses after a longer IM treatment. This may occur in patients in whom the dose of IM was reduced during the treatment for various reasons and who therefore did not achieve CCyR or MMR in defined optimal time.

## Conclusion

Our data, which are highly comparable to clinical trials or single-center intention-to-treat analysis, significantly show the effectiveness of IM as a first-line treatment in patients with CP-CML. The response criteria and their predictive role defined by ELN were helpful at given time points; however, the ELN 2009 did not significantly alter the prognostic accuracy compared with ELN 2006. Additionally, the powerful value of cytogenetic response achievement at the 6th and 12th months was proved for outcome prognostication. Moreover, PFS and EFS with more detailed definitions in comparison with most of other studies were improved, with deeper molecular response including MMR at 12 months. The cumulative incidences of CCyR and MMR were significantly associated with the levels of BCR-ABL transcripts in the 3rd month. We should expect a significant impact of molecular response at 3 months on survivals, which we did not confirm. To prove whether the BCR-ABL transcript level cut offs at the 3rd month landmark have significant impact on better outcome remains a challenge for our forthcoming study that will require a larger cohort of patients and longer follow-up.

*Authorship:* H. K. and K. M. P. – conception and design, data interpretation, manuscript writing; JMuz – data evaluation, statistical analysis, revisions; E. F., Z. R., and M. M. S. – clinical data provision, critical revisions, and valuable recommendations; D. Z., E. D., Z. M. S., J. V., L. D., E. M., E. T., J. Ch., I. M., E. C., V. K., M. K. – clinical data provision; K. M. and M. J. – cytogenetic data provision; K. M. P., J. Mor., D. D. – molecular data provision; L. D. – revisions, data evaluation, and statistical analysis; P. C., M. T., and K. I. – contribution to study conception and design, critical revisions.

## Conflict of Interest

H. K., K. M. P., M. K., E. F., J. V., K. I., Z. R., D. Z., E. C. – advisory board – BMS and Novartis. K. M. P. – research supports – BMS, Novartis, Roche.

## Supporting Information

Figure S1 Cumulative incidence of (a) CHR, MCyR, CCyR (*N* = 458), (b) MMR and CMR (*N* = 199). CHR, complete hematologic responses; MCyR, major cytogenetic response; CCyR, complete cytogenetic response; MMR, major molecular response; CMR, complete molecular response.

**Table S1.** The cumulative incidence of survival (*n* = 458). **Table S2.** The cumulative incidence of responses. **Table S3.** Cumulative incidence of CCyR (A) and MMR (B) according to BCR-ABL level in 3rd month. CCyR, complete cytogenetic response; MMR, major molecular response.
