# Supplementary material for: Evaluation of 5-year imatinib treatment of 458 patients with CP-CML in routine clinical practice and prognostic impact of different BCR-ABL cutoff levels
Source: Cancer Med. 2013 Feb 21;2(2):216–25. doi: 10.1002/cam4.59 (PMC3639660; doi:10.1002/cam4.59)
Supplement: Figure S1 — Cumulative incidence of (a) CHR, MCyR, CCyR (N = 458), (b) MMR and CMR (N = 199). CHR, complete hematologic responses; MCyR, major cytogenetic response; CCyR, complete cytogenetic response; MMR, major molecular response; CMR, complete molecular response. [file cam40002-0216-sd1.pptx]

## Slide 1
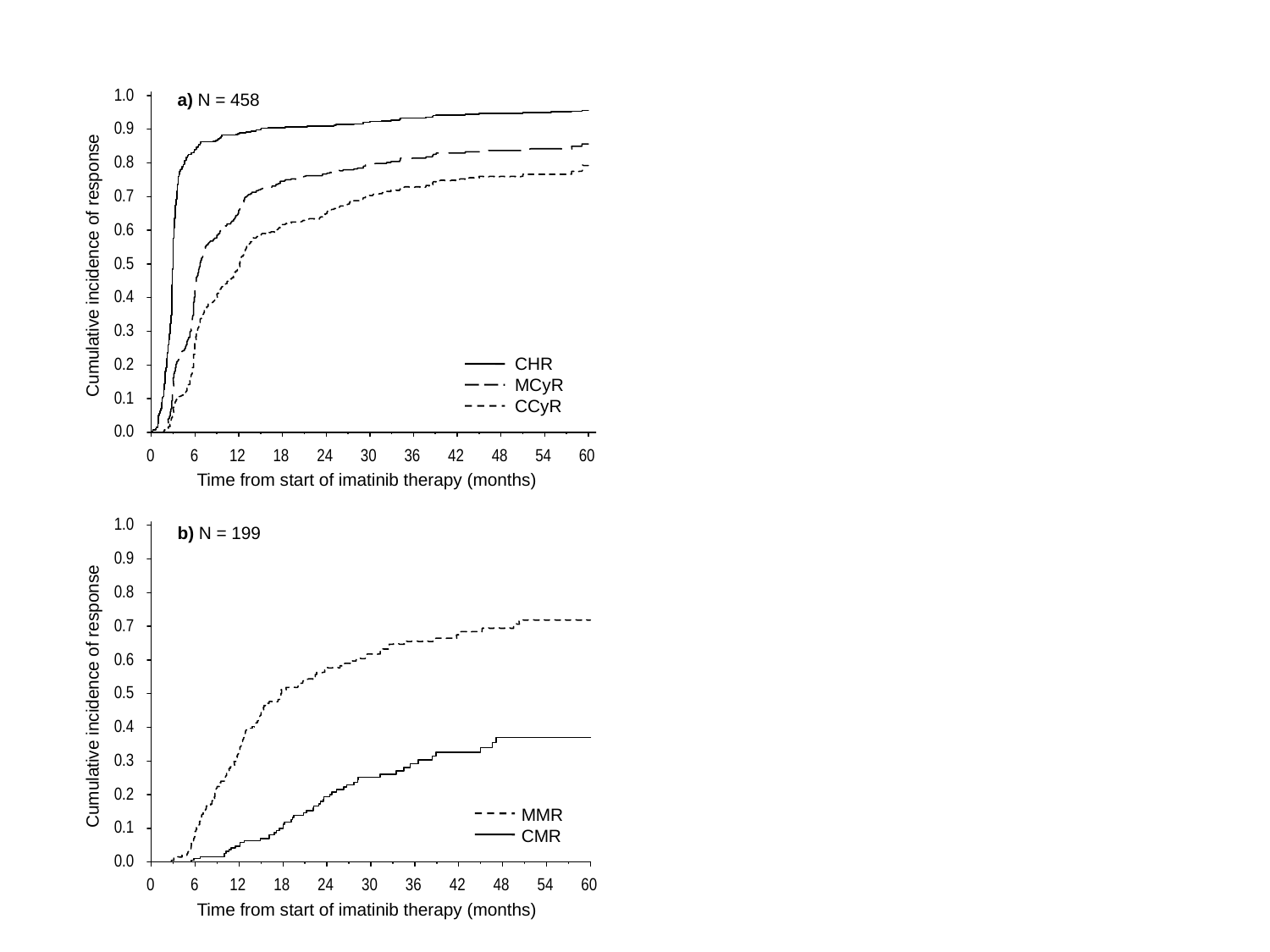

a) N = 458
Cumulative incidence of response
CHR
MCyR
CCyR
Time from start of imatinib therapy (months)
b) N = 199
Cumulative incidence of response
MMR
CMR
Time from start of imatinib therapy (months)
