# Supplementary material for: Evaluation of 5-year imatinib treatment of 458 patients with CP-CML in routine clinical practice and prognostic impact of different BCR-ABL cutoff levels
Source: Cancer Med. 2013 Feb 21;2(2):216–25. doi: 10.1002/cam4.59 (PMC3639660; doi:10.1002/cam4.59)
Supplement: Supplementary file 2 — Table S1. The cumulative incidence of survival (n = 458). Table S2. The cumulative incidence of responses. Table S3. Cumulative incidence of CCyR (A) and MMR (B) according to BCR-ABL level in 3rd month. CCyR, complete cytogenetic response; MMR, major molecular response. [file cam40002-0216-sd2.docx]

Supplement Table 1. The cumulative incidence of survival (n = 458)

| **Time (months)** | **OS**  % (95% CI) | **OS_CML_**  % (95% CI) | **TFS**  % (95% CI) | **PFS**  % (95% CI) | **EFS**  % (95% CI) | **ATFS**  % (95% CI) |
| --- | --- | --- | --- | --- | --- | --- |
| **6** | 99.6 (99.0; 100) | 100 | 99.3 (98.6; 100) | 98.2 (96.9; 99.4) | 92.7 (90.3; 95.1) | 95.2 (93.2; 97.2) |
| **12** | 98.2 (97.0; 99.4) | 99.3 (98.6; 100) | 98.8 (97.8; 99.8) | 95.5 (93.5; 97.5) | 84.1 (80.7; 87.6) | 91.9 (89.4; 94.4) |
| **18** | 96.3 (94.5; 98.1) | 98.6 (97.4; 99.7) | 97.2 (95.6; 98.8) | 91.2 (88.3; 94.0) | 76.6 (72.6; 80.6) | 86.6 (83.4; 89.8) |
| **24** | 94.6 (92.4; 96.8) | 97.4 (95.8; 99.0) | 96.9 (95.2; 98.6) | 88.9 (85.7; 92.1) | 73.4 (69.1; 77.7) | 82.1 (78.4; 85.8) |
| **30** | 94.0 (91.6; 96.3) | 97.4 (95.8; 99.0) | 95.8 (93.6; 97.9) | 87.2 (83.7; 90.6) | 70.0 (65.4; 74.6) | 79.9 (76.0; 83.9) |
| **36** | 92.8 (90.1; 95.5) | 96.6 (94.6; 98.5) | 95.3 (93.0; 97.6) | 86.7 (83.1; 90.3) | 69.6 (64.9; 74.2) | 77.2 (72.9; 81.5) |
| **42** | 91.7 (88.6; 94.7) | 96.6 (94.6; 98.5) | 93.9 (90.9; 96.9) | 84.7 (80.5; 88.8) | 68.3 (63.4; 73.2) | 74.0 (69.2; 78.8) |
| **48** | 91.1 (87.8; 94.3) | 96.6 (94.6; 98.5) | 93.9 (90.9; 96.9) | 83.2 (78.6; 87.7) | 66.0 (60.6; 71.4) | 69.7 (64.2; 75.2) |
| **54** | 90.2 (86.5; 93.8) | 96.6 (94.6; 98.5) | 93.9 (90.9; 96.9) | 80.7 (75.2; 86.3) | 62.5 (56.1; 68.9) | 65.7 (59.3; 72.2) |
| **60** | 90.2 (86.5; 93.8) | 96.6 (94.6; 98.5) | 93.9 (90.9; 96.9) | 80.7 (75.2; 86.3) | 58.8 (49.6; 68.0) | 61.8 (53.7; 69.9) |

Supplement Table 2. The cumulative incidence of responses

|  | **N = 458** | | | **N = 199** | |
| --- | --- | --- | --- | --- | --- |
| **Time (months)** | **CHR**  % (95% CI) | **MCyR**  % (95% CI) | **CCyR**  % (95% CI) | **MMR**  % (95% CI) | **CMR**  % (95% CI) |
| **6** | 83.8 (80.5; 87.2) | 42.6 (38.0; 47.1) | 27.1 (23.0; 31.1) | 9.1 (5.1; 13.1) | 1.0 (0; 2.4) |
| **12** | 88.7 (85.8; 91.6) | 66.0 (61.6; 70.3) | 49.1 (44.5; 53.7) | 32.6 (25.9; 39.2 | 4.7 (1.7; 7.7) |
| **18** | 90.5 (87.8; 93.2) | 74.6 (70.5; 78.6) | 61.7 (57.2; 66.2) | 51.2 (44.0; 58.5) | 11.3 (6.6; 16.0) |
| **24** | 90.9 (88.3; 93.6) | 76.7 (72.8; 80.6) | 64.9 (60.5; 69.4) | 57.0 (49.7; 64.3) | 19.4 (13.3; 25.5) |
| **30** | 92.3 (89.8; 94.7) | 79.5 (75.7; 83.3) | 70.3 (65.9; 74.6) | 61.8 (54.5; 69.1) | 25.2 (18.3; 32.1) |
| **36** | 93.4 (91.1; 95.7) | 81.4 (77.7; 85.1) | 72.9 (68.6; 77.2) | 65.5 (58.2; 72.8) | 29.2 (21.6; 36.7) |
| **42** | 94.3 (92.1; 96.4) | 82.9 (79.2; 86.6) | 74.8 (70.5; 79.1) | 67.5 (60.1; 74.8) | 32.6 (24.5; 40.8) |
| **48** | 94.7 (92.6; 96.8) | 83.7 (80.0; 87.3) | 76.0 (71.7; 80.3) | 69.4 (62.1; 76.7) | 37.0 (28.0; 45.9) |
| **54** | 94.9 (92.9; 97.0) | 84.2 (80.6; 87.9) | 76.6 (72.3; 81.0) | 71.8 (64.4; 79.3) | 37.0 (28.0; 45.9) |
| **60** | 95.6 (93.7; 97.5) | 85.6 (82.0; 89.3) | 79.2 (74.7; 83.7) | 71.8 (64.4; 79.3) | 37.0 (28.0; 45.9) |

Supplement Table 3. Cumulative incidence of CCyR (A) and MMR (B) according to BCR-ABL level in 3^rd^ month

| **A: Cumulative incidence of CCyR in % (CI)** | | | |
| --- | --- | --- | --- |
| **Time**  **(months)** | **BCR-ABL level in 3^rd^ month** | | |
|  | **≤ 1 %** | **> 1 % and ≤ 10 %** | **> 10 %** |
| **6** | 25.7 (11.0; 40.4) | 34.0 (21.1; 46.9) | 5.3 (0; 11.1) |
| **12** | 62.9 (46.4; 79.3) | 69.9 (57.3; 82.6) | 25.4 (13.7; 37.0) |
| **18** | 79.1 (64.2; 93.9) | 76.1 (64.2; 88.1) | 51.3 (37.4; 65.1) |
| **24** | 86.5 (73.1; 100) | 76.1 (64.2; 88.1) | 53.6 (39.6; 67.6) |
| **30** | 86.5 (73.1; 100) | 82.7 (71.7; 93.7) | 62.0 (47.4; 76.6) |
| **36** | 86.5 (73.1; 100) | 82.7 (71.7; 93.7) | 65.3 (50.6; 80.1) |
| **42** | 86.5 (73.1; 100) | 85.3 (74.6; 96.0) | 65.3 (50.6; 80.1) |
| **48** | 86.5 (73.1; 100) | 85.3 (74.6; 96.0) | 65.3 (50.6; 80.1) |
| **54** | - | - | 65.3 (50.6; 80.1) |
| **60** | - | - | 65.3 (50.6; 80.1) |
| **B: Cumulative incidence of MMR in % (CI)** | | | |
| **Time**  **(months)** | **BCR-ABL level in 3^rd^ month** | | |
|  | **≤ 1 %** | **> 1 % and ≤ 10 %** | **> 10 %** |
| **6** | 25.7 (11.0; 40.4) | 11.3 (2.7; 19.9) | 5.3 (0; 11.3) |
| **12** | 69.8 (53.7; 85.9) | 36.8 (23.5; 50.2) | 19.0 (8.2; 29.7) |
| **18** | 85.2 (72.3; 98.2) | 61.0 (47.2; 74.8) | 34.4 (20.8; 48.0) |
| **24** | 85.2 (72.3; 98.2) | 70.4 (57.0; 83.7) | 39.7 (25.2; 54.1) |
| **30** | 85.2 (72.3; 98.2) | 72.7 (59.6; 85.8) | 51.3 (35.7; 67.0) |
| **36** | - | 77.9 (65.3; 90.5) | 58.9 (42.3; 75.5) |
| **42** | - | 83.1 (71.4; 94.8) | 58.9 (42.3; 75.5) |
| **48** | - | 85.7 (74.5; 96.8) | 58.9 (42.3; 75.5) |
| **54** | - | - | 58.9 (42.3; 75.5) |
| **60** | - | - | 58.9 (42.3; 75.5) |
